# Bacteriophages: Uncharacterized and Dynamic Regulators of the Immune System

**DOI:** 10.1155/2019/3730519

**Published:** 2019-09-08

**Authors:** Anshul Sinha, Corinne F. Maurice

**Affiliations:** Department of Microbiology & Immunology, McGill University, Montreal, QC, Canada

## Abstract

The human gut is an extremely active immunological site interfacing with the densest microbial community known to colonize the human body, the gut microbiota. Despite tremendous advances in our comprehension of how the gut microbiota is involved in human health and interacts with the mammalian immune system, most studies are incomplete as they typically do not consider bacteriophages. These bacterial viruses are estimated to be as numerous as their bacterial hosts, with tremendous and mostly uncharacterized genetic diversity. In addition, bacteriophages are not passive members of the gut microbiota, as highlighted by the recent evidence for their active involvement in human health. Yet, how bacteriophages interact with their bacterial hosts and the immune system in the human gut remains poorly described. Here, we aim to fill this gap by providing an overview of bacteriophage communities in the gut during human development, detailing recent findings for their bacterial-mediated effects on the immune response and summarizing the latest evidence for direct interactions between them and the immune system. The dramatic increase in antibiotic-resistant bacterial pathogens has spurred a renewed interest in using bacteriophages for therapy, despite the many unknowns about bacteriophages in the human body. Going forward, more studies encompassing the communities of bacteria, bacteriophages, and the immune system in diverse health and disease settings will provide invaluable insight into this dynamic trio essential for human health.

## 1. Introduction

The human gut is a dense and diverse ecosystem containing a collection of trillions of bacteria, archaea, viruses, and eukaryotic microorganisms, collectively termed the gut microbiota. Advances in single-cell techniques, animal models, and “omics” approaches to study the human gut microbiota have unveiled the role of these commensal microorganisms as an active component of human physiology and health. Indeed, the gut bacterial community expands human metabolism by providing its host with metabolic pathways involved in breaking down otherwise indigestible nutrients and xenobiotics, compounds foreign to a living organism [1, 2]. The gut microbiota also protects against the invasion of pathogens by occupying all available niches in the gut and producing inhibitory compounds preventing the colonization of the gut by these and other microorganisms [3, 4]. Furthermore, the development of a mature immune system has been tied to bacterial colonization of the infant gut [5, 6].

Several genetic and environmental factors shape the composition of the gut microbiota. As such, a number of human diseases, including inflammatory bowel diseases (IBD), obesity, allergies, and diabetes, have all been associated with disease-specific shifts in gut microbial communities [7–12]. Despite the tremendous recent advances in this field, most studies on the gut microbiome remain incomplete, as they do not consider one of the main agents of bacterial death and horizontal gene transfer in nature, namely, bacteriophages (phages) [13]. For example, it is estimated that up to 50% of bacterial mortality in the oceans worldwide is due to daily phage infection and a selection of human bacterial pathogens, such as *Vibrio cholerae*, acquires their pathogenicity through phage-encoded toxins [14–16]. In the gut, these bacteria-specific viruses are estimated to be as abundant as their bacterial hosts and constitute a source of polysaccharide and carbohydrate metabolism genes and antibiotic resistance, as well as cofactors that increase bacterial growth and fitness [13, 17–19]. Yet, their interactions with their bacterial hosts and the human immune system remain poorly described.

Phages were first discovered in 1915 by Twort and independently rediscovered and named in 1917 by d'Herelle, who named them after their lethal mode of action on bacteria (bacteriophage means “bacteria eater”) [20, 21]. Both researchers studied phages in attempts to use them to cure bubonic plague or cholera, but their unsuccessful attempts and the concomitant discovery of antibiotics in the 1940s led to the widespread abandon of phages for therapy, except in Russia, Georgia, and Poland [22]. Despite this, phages remained studied in the laboratory context, where they have been instrumental for the development of molecular biology [23]; in aquatic systems, where they have been shown to play major roles in biogeochemical cycles [24, 25]; and in the food industry to control food-borne pathogens [26]. With the recent and dramatic increase in antibiotic resistance, phages have returned to the spotlight as a promising therapeutic tool, despite the many unknowns about their roles in the human body. After an overview of phage communities in the human gut during human development, we then detail their effects on the immune response through their actions on their bacterial targets and summarize the recent evidence for direct interactions between them and the immune system. Finally, we conclude with opportunities and challenges these interactions can represent in the context of phage therapy.

## 2. Bacteriophages in the Human Gut: Diversified, Numerous, and Uncharacterized

Despite advancements in high-throughput sequencing technologies, the characterization of phages in the human gut remains limited, mostly due to difficulties in phage isolation and genome annotation [27]. The inherent mosaic nature of phage genomes, their small size (approx. 30 kb in the gut [28]), and absence of universal genetic markers make annotation of phages challenging. Regardless, recent characterizations of the collection of phage genes (i.e., the phageome) have led to better identification of phages in the mammalian gut in health and disease, shedding some light on the compositional and functional diversity of these entities [29].

### 2.1. Phage Communities in the Healthy Human Gut

Phage sequences dominate the viral sequences detected in the human gut (the gut virome), despite most of the phage sequences corresponding to “dark matter” remaining to be characterized [27]. Within the characterized phages in the gut, the tailed dsDNA phages of the *Caudovirales* order are the most abundant, composed of the *Myoviridae*, *Podoviridae*, and *Siphoviridae* families, followed by the ssDNA *Microviridae* phage family [19, 30]. As RNA phages are currently considered to be transient members of the gut originating from our diet [31], most of our discussion here will focus on DNA phages. Phage diversity typically follows that of the main bacterial hosts in the gut, namely, the Firmicutes, Bacteroidetes, Proteobacteria, and Actinobacteria [32, 33], even during the transitions from childhood to adulthood.

**Figure 1:**
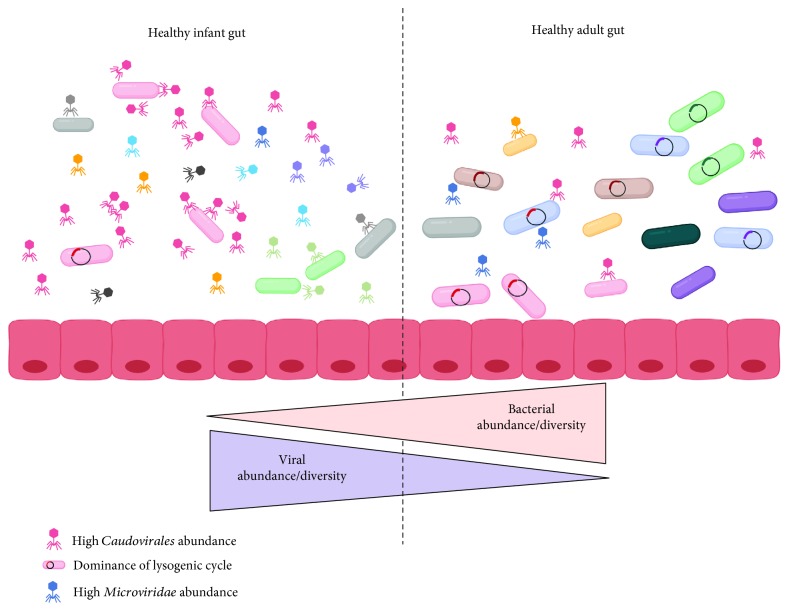
Characteristics of phage-host dynamics in the healthy infant and adult gut. During the first 2-3 years of life, there are drastic changes in the bacterial and phage communities in the healthy gut. Kill the Winner dynamics dominate during childhood, resulting in lytic replication and high phage abundance and diversity, particularly within the phage order *Caudovirales* (red). Piggyback the Winner dynamics are hypothesized to be prevalent in the healthy adult gut, where an increase in lysogenic replication coincides with a decrease in overall phage abundance and diversity. The abundance of *Microviridae* (blue) increases, and the phage community remains relatively stable over time. An absence of phage predation may lead to the expansion of bacterial abundance and diversity observed in the adult gut. Image created using BioRender.

Phages have been detected at low levels in newborns shortly after birth and are suggested to be from maternal and environmental origins [34, 35]. Within 2 weeks of life, phage communities go through drastic changes in their diversity and abundances in the infant gut [35]. Characterization of the viromes from mother-infant pairs suggests that breast milk may be an important initial source of phages in the infant gut [35–38]. Until approximately 2 years of age, the bacterial communities in the gut follow rapid expansions in their numbers and diversity (Figure 1) [39, 40]. Initially, this is also the case for the phage communities, but they rapidly contract and decrease in diversity with age (Figure 1) [34]. The rich collection of different *Caudovirales* phages found in the first few months of life decreases and seems to be replaced by the *Microviridae* species (Figure 1) [34]. The mechanisms underlying this dichotomy between bacterial and phage communities remain unclear, as not all shifts in phage diversity reflect the bacterial shifts. However, as we further detail, this could be driven in part by changes in phage replication cycles. Interestingly, one year after birth, phage communities were still different between children born vaginally and through C-section, despite their gut bacterial communities being similar, highlighting the importance of vertical transmission for some phage taxa [41].

From early childhood into adulthood, phage communities in the gut are unique to each individual, as demonstrated by the study of monozygotic and dizygotic twin pairs [32]. Similar to gut bacterial communities, relatives and unrelated household members share more phages than unrelated individuals [32], but each individual harbours a unique phage signature. There is increasing evidence for clusters of phage species that are shared among many healthy individuals, which include the ubiquitous *crAssphage* [19, 42, 43]. Approximately 40% of phages in these clusters are not found in adults with IBD, suggesting that these phages could be important biomarkers of health [19], yet these phages represent only a fraction (<5%) of the estimated phage diversity in the gut [42, 44]. More studies characterizing gut phage communities in adults from a variety of locations and diet are thus warranted to better understand the roles of these phages as markers of health. In the gut of healthy adults, phage communities remain relatively stable over time, with 80% of the same phage sequences detected in a given individual for 2.5 years [32, 42]. Unlike other ecosystems, the abundance of phages relative to their bacterial hosts, determined with the virus-to-bacteria ratio (VBR), is low and between 0.1 : 1 and 1 : 1. This suggests a dominance of the lysogenic replication cycle over the lytic cycle in the healthy adult gut, and as detailed below, there is increasing evidence linking disease with modifications of phage replication cycles.

### 2.2. Phage Replication Strategies and Implications for Development and Health

Phages replicate mostly through the lytic or lysogenic replication cycles, which have been extensively described elsewhere [24, 44]. In brief, the lytic cycle is characterized by the direct production of new phages after infection of a bacterial cell, causing bacterial cell death. Lysogeny is characterized by the integration of the phage genome into the bacterial genome or maintained as a plasmid. The integrated phage, or prophage, remains in its bacterial host until induction occurs, triggering a return to the lytic production of new phages [44]. It is currently considered that phages in the gut of infants up to 24 months old replicate through the lytic cycle, as both bacterial and phage communities are highly dynamic and go through drastic changes in abundances and composition [34, 44]. During this developmental period, phages are suggested to alter bacterial populations and maintain high levels of bacterial diversity through “Kill the Winner (KtW)” dynamics [34, 45, 46]. In these predator-prey interactions, phage infection controls the abundance of the dominant members of the bacterial community.

In contrast, phages in the gut of healthy adults seem to be integrated prophages, leading to the dominance of the lysogenic cycle (Figure 1). This is supported by the low VBRs, stability of phage abundance and diversity, absence of KtW dynamics, and the abundance of phages classified as temperate based on sequence homology and the presence of the *integrase* gene necessary for genome integration into the bacterial host [32, 33, 44]. The lysogenic cycle is typically found in low-nutrient and low bacterial abundance settings, which are not prevailing conditions in the gut. The prevalence of lysogeny despite the high abundance of actively replicating bacteria in the gut has led to the “Piggyback the Winner (PtW)” model, whereby phages may undergo lysogenic replication in such conditions to take advantage of the high fitness of their bacterial hosts [47]. In extension of this idea, it is hypothesized that there is a gradient of lysogenic to lytic replication across the gut mucus layer. In the lumen and the top mucus layer, where the bacterial load is higher, lysogenic replication dominates in agreement with PtW dynamics; while in the inner mucus layer, with lower bacterial load, lytic replication dominates [47]. Diseases where the mucosal layer is disrupted could thus lead to more lytic replication, further enhancing the changes in bacterial communities and associated pathologies.

**Figure 2:**
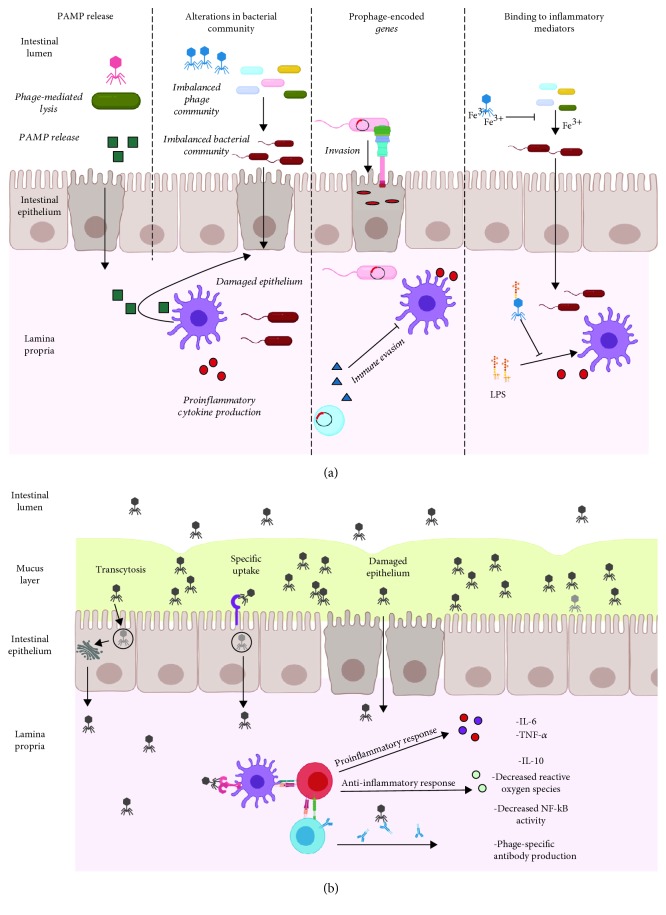
Crosstalk between phages and the immune system. (a) Indirect influences on immune responses. Phage infection may lead to the release of PAMPs, which can translocate the gut epithelium and induce proinflammatory responses. In the case of imbalanced phage communities, infection of certain bacterial species may lead to an altered microbiota, overgrowth of pathogens, and chronic inflammation. Prophage-encoded genes can aid pathogens in their abilities to damage and invade the epithelium and evade the immune system by directly inhibiting phagocytic cells. Sequestration of iron by phage tail domains could prevent pathogen overgrowth in the intestines. Binding of LPS by phage head proteins may dampen LPS-induced inflammation. (b) Direct stimulation of immune responses. Phages may cross the intestinal epithelium in 3 ways: nonspecific transcytosis, specific recognition of eukaryotic cells via structures that resemble bacterial receptors, and passage through damaged epithelial cells with defects in permeability. Once in the lamina propria, phages can interact with the intestinal immune system to generate pro- or anti-inflammatory responses and generate specific antiphage-neutralizing antibodies. The image was created using BioRender.

Interestingly, metagenomic studies report that most detected prophage sequences in the human and murine gut are integrated within bacteria from the Firmicutes phylum [32, 33, 42, 48]. This could have strong implications for human health, as the diversity and abundance of bacterial taxa within the Firmicutes are typically altered and possibly implicated in a variety of diseases [49]. The ubiquity of phages in the gut and their ability to modulate bacterial communities in other ecosystems suggest that they could be active players in human health and interact with the host immune system. Several immunological diseases, including inflammatory bowel diseases (IBD), Parkinson's disease, and Type 1 and Type 2 diabetes, have been associated with alterations of the gut phage community [50–54]. Understanding the direct and indirect ways by which phages interact with the immune system, as summarized in Figure 2, will help us gain insight into the functional role that these viruses play in human health and disease.

## 3. Bacterial-Mediated Interactions between Phages and the Immune System

As previously detailed, phage communities are specific to their bacterial hosts and can alter bacterial diversity and metabolism in a number of ways: by undergoing different replication cycles, infecting different bacterial hosts, carrying unique suites of genes augmenting host fitness, and having distinct binding properties. Given the many intricate interactions between the immune system and our resident bacterial communities, phages could be indirectly influencing these interactions by manipulating their hosts.

### 3.1. The Intestinal Bacterial Community and the Immune System

In order to understand how phage-mediated changes in the gut microbiota can influence immunity, it is important to consider the interactions between bacteria and the immune system. The bacterial component of the microbiota has been heavily implicated in the development of immune cells and the regulation of immune responses [55]. Initial exposure to microbial products is important in developing tolerance to commensals [56, 57]. In addition, the development of isolated lymphoid follicles, secretion of IgA, and maturation and homeostasis of CD4+ T cells and invariant natural killer T cells have all been tied to early exposure to microbes or microbial products [58–61]. The commensal bacterial community also plays an important role in the regulation of immune responses. For instance, various *Clostridia* species from the clusters IV and XIVa have been shown to induce mucosal regulatory T cell (Treg) accumulation and IL10 production, central to dampening proinflammatory immune responses [62, 63]. Many of these regulatory interactions can be linked to the production of short chain fatty acids (SCFAs), often produced by microbial fermentation of diet-derived fibres [64].

The intestinal bacterial communities also play an important role in preventing the colonization and systemic dissemination of potentially pathogenic enteric microbes [65–68]. The outgrowth of these pathogens, often belonging to the Proteobacteria phyla, has been associated with inflammatory diseases, with evidence indicating that some of these microorganisms can thrive in an inflamed environment [69–72]. It has been suggested that the increase in abundance of pathogens with increased inflammatory capabilities could trigger a feedback loop, whereby the proliferation of pathogenic organisms leads to increased inflammation and an environment that further selects for pathogen dissemination [55]. Consequently, a number of immunological disorders have been associated with shifts in microbial community composition [10, 73, 74]. We are now beginning to gain some insight into how phages might be driving these changes.

### 3.2. Phage-Mediated Alterations in the Intestinal Bacterial Communities: Implications for Immune Disorders

Despite the prevalence of lysogeny in the gut, there is growing evidence that phage predation can shape microbial communities in this environment [75–79]. Reyes et al. staged a “phage attack” of isolated virus-like particles (VLPs) from the feces of 5 unrelated volunteers to germ-free mice colonized with a collection of 15 bacterial isolates. Following phage administration, changes in the relative abundance of members of the bacterial community could be detected, suggesting that gut-derived phages were still infectious [75]. Using a similar approach, Hsu et al. colonized germ-free mice with a mock community of 10 known bacterial isolates before administering phages specific to a subset of these bacteria. They concluded that phage predation had cascading effects on the microbiota due to knockdown of susceptible species and subsequent disturbances to networks of interbacterial interactions. Further, these phage-induced changes of the microbiota were sufficient to alter the concentrations of a number of bacterial-derived metabolites, including neurotransmitters, amino acids, and bile salts [77].

These phage-mediated changes of gut bacterial communities could have downstream effects on immune signaling by allowing for the proliferation of proinflammatory or pathogenic microorganisms or altering the production of immunomodulatory bacterial-derived products (Figure 2(a)). The detection of bacterial DNA systemically following oral phage administration supports the idea that phage-mediated cell lysis could be responsible for the release of immunostimulatory pathogen-associated molecular patterns (PAMPs) [80]. With increased gut permeability, these PAMPs could translocate the epithelial layer and cause immune activation (Figure 2(a)) [80].

Both phage and bacterial communities have been shown to be altered in the context of intestinal inflammation [10, 50, 51, 81, 82]. Norman et al. concluded that the increase in *Caudovirales* and the expansion of overall phage richness observed in IBD patients were not driven by increases in bacterial richness [50]. The authors also found significant associations between the expansion of *Caudovirales* and specific members of the bacterial community [50]. These findings suggest that changes in the bacterial community associated with IBD could be driven by an imbalance of phages infecting these bacteria. In line with this hypothesis, Cournault et al. found that phages which infect the bacterium *Faecalibacterium prausnitzii* were elevated in the feces of IBD patients [83]. Since levels of *F. prausnitzii*, a producer of the SCFA butyrate, are depleted in the gut of IBD patients, the expansion of phages infecting these bacterial taxa could contribute to its loss and increased inflammation during the course of disease [84]. Similar associations have been made in Parkinson's disease (PD), where the gut microbiota has been implicated in disease progression through the regulation of inflammatory responses and subsequent interactions with the enteric nervous system [85–88]. In PD patients, there is an increase in lytic *Lactococcus* phages and a corresponding decrease in *Lactococcus* bacteria, which have been shown to be potent inducers of anti-inflammatory responses and involved in the production of neurotransmitters [52]. Most recently, Tetz et al. found that children who presented seroconversion or developed Type 1 diabetes (T1D) had a high abundance of lysogenic *E. coli* phages compared to their bacterial hosts [54]. Interestingly, these data could suggest that prophage induction could cause release of DNA-amyloid complexes and trigger autoimmune cascades leading to T1D development [54].

The findings mentioned above show clear associations between altered phage and bacterial communities, and inflammatory diseases. Additional studies will need to identify factors that influence the changes in phage communities during disease. Different diets and specific dietary components have now been shown to shape the intestinal phage communities and the phageome [33, 89, 90]. Xenobiotics have also been shown to increase the expression of prophage induction genes, which could have widespread effects on bacterial and phage community composition [91]. Given that KtW or predator-prey interactions between phages and their hosts are most prevalent in early childhood, the infant phageome may be key in driving the appropriate maturation of the gut microbiota. Understanding the factors that shape the initial phage community during early childhood will provide insight into how microbial imbalances and their associated inflammatory diseases develop.

### 3.3. Phage-Encoded Genes Involved in Crosstalk with the Immune System

Beyond regulating the diversity, abundance, and metabolism of bacterial communities, phages are also powerful agents of horizontal gene transfer between bacteria. Prophages integrated into bacterial chromosomes or maintained as plasmids within bacterial cells account for important genetic differences between strains of the same species [92, 93]. In a process known as lysogenic conversion, genes within these integrated prophages can confer a fitness advantage to their bacterial host [94]. Many of these phage-encoded genes are involved in “superinfection exclusion,” where integrated prophages are involved in preventing their bacterial host from further infection by closely related phages [95, 96]. Importantly, several genes carried by prophages have been found to increase the pathogenic potential of their host, either through the expression of phage-encoded virulence factors or other proteins that assist in immune evasion (Figure 2(a)). Thus, the genetic material that prophages provide to their lysogens has strong implications for how the immune system responds to, or can control, certain members of a microbial community.

Prophage-encoded toxins can be found in several unrelated bacterial species. Enterohemorrhagic *E. coli* (EHEC), *Clostridium botulinum*, *C. difficile*, *Vibrio cholerae*, and *Streptococcus pyogenes*, among others, rely on genetic material provided by prophages to produce toxins or proteins that regulate toxin production [97–100]. In *C. difficile* infections specifically, toxin B causes increased IL-8 production and immune-mediated damage of the intestinal epithelium [101]. *C. difficile* prophages do not encode this toxin [99]; however, lysogeny of several strains can increase its levels, suggesting a mechanism where phage integration could drive toxin B production and downstream proinflammatory responses [99]. Other phage-encoded genes, which are not toxins, may assist the invasive properties of enteric pathogens. *Salmonella typhimurium* expresses the rho GTPase, *sopE*, which is derived from the SopE*φ* temperate phage [102]. SopE is secreted into host cells via a type 2 secretion system and aids the entry of the bacterium by inducing membrane ruffling (Figure 2(a)) [103]. Delivery of SopE into stromal cells has also been shown to elicit mucosal inflammatory responses via caspase-1 activation and contribute to murine colitis [104, 105]. In turn, gut inflammation can accelerate the transfer of *sopE* between *Salmonella* strains through activation of the SOS stress response and subsequent prophage induction [106]. Some bacteria use prophage-encoded genes to evade the immune system to aid in their dissemination. For instance, *Staphylococci* prophages contain several genes involved in immune evasion, which integrate within the *β*-haemolysin gene [107]. The prophage-encoded chemotaxis inhibitory protein (CHIPS) and the Staphylococcal complement inhibitor (SCIN) block complement activation and neutrophil-mediated killing [108]. The Panton-Valentine leukocidin, which has been associated with methicillin-resistant *Staphylococcus aureus* (MRSA), can directly inhibit phagocytes by forming pores in the membranes of these cells [109, 110]. Collectively, these studies demonstrate that phage-encoded genes can have a diverse and profound influence on the interactions between bacteria and the immune system.

### 3.4. Phage Binding to Inflammatory Mediators

The exposed phage protein coat and tail fibres provide opportunities for unique binding sites between phages and their direct environment. Most studied interactions focus on phage binding to receptors on the surface of bacterial cells and subsequent infection [111, 112]. However, there is increasing evidence that the binding properties of phages and their associated functions are more complex. Structural analysis of the tail fibre region in T4 phages revealed that the needle domain contains 7 iron ions coordinated by histidine residues [113]. Iron binding has now been associated with several phages (Figure 2(a)) [114, 115]. Interestingly, Penner et al. found that the Pf4 phage could sequester Fe^3+^ and subsequently inhibit the formation of *Aspergillus fumigatus-*associated biofilms [115]. Increases in the amount of free iron have similarly been associated with increased risk of infection, virulence, and the outgrowth of pathogens including *V. vulnificus*, *S. typhimurium*, and *Yersinia* species [116–119]. Phages can also alter immune responses by directly binding to inducers of inflammation: for example, the tail adhesin gp12 has been shown to mediate adsorption of T4 phages to *E. coli* cells [120]. More recently, Miernikiewicz et al. built on these findings to show that recombinant gp12 could not only bind to LPS but could also prevent LPS-induced production of proinflammatory cytokines in mice (Figure 2(a)) [121].

The ubiquity of phage-mediated binding of LPS and iron sequestration in the gut remains unclear, and other mechanisms could also be taking place. As we better characterize and annotate the phages in the human gut, we will gain a greater appreciation for how phage-mediated binding interactions might modulate inflammatory responses. Studying the immune response to both bacterial and phage communities in the gut will unveil many underlying interactions between these three parties, with some studies already demonstrating direct crosstalk between phages and the immune system.

## 4. Direct Crosstalk between Bacteriophages and the Immune System in the Gut

Phages are unable to infect eukaryotic cells, mostly due to differences between prokaryotic and eukaryotic replication and transcriptional machinery. Still, the human body is under constant exposure to diverse and abundant phage communities. Phages have been found in the gut, skin, lung, and bloodstream and have even been detected in cerebrospinal fluid and *in utero* following systemic dissemination. Understanding how phages access these disparate sites and how they interact with the mammalian immune system has important implications for human health and disease.

### 4.1. Crossing the Epithelial Barrier

In the mucosal layer above the epithelium, phage abundance has been shown to be over four times higher than the adjacent luminal area in a number of metazoan species [122]. The presence of phages systemically in several mammalian species suggests that the phages found in the mucosal layer can cross the epithelial cell layer and interact with underlying immune cells. Tight junctions between epithelial cell layers prevent passage of molecules greater than 0.4 nm, which includes phages [123]. It was thus suggested that the most probable mode of transportation of phages through this layer would be when the epithelium is compromised. In this case, a loss in tight junction functionality, responsible for tight cell-cell adhesion, may cause points of entry for phages (Figure 2(b)). Yet, phages have been detected in humans and rodents without any deficiencies in intestinal permeability, suggesting alternative pathways by which phages cross the epithelium [124–128].

In one example of phages interacting with mammalian cells, Lehti et al. described that phages could be internalized by eukaryotic cells by binding to moieties that resemble bacterial phage receptors (Figure 2(b)) [129]. Here, the *Escherichia coli* phage PK1A2 was shown to be internalized by neuroblastoma cells, which contain surface polysialic acid that are identical in structure to the bacterial K1 polysialic acid capsule [129, 130]. While phage DNA was shown to be degraded in the lysosome, this suggests that molecular mimicry could allow for direct interactions between phages and eukaryotic cells. Similarly, several groups have expressed eukaryotic surface structures on phage capsids to enter various eukaryotic cells for gene delivery [131]. Namdee et al. demonstrated this in the gut using a filamentous phage expressing an integrin binding motif [132]. Another and more nonspecific mechanism of phage uptake was described by Nguyen et al. (Figure 2(b)) [133]. The authors used an *in vitro* transwell system to measure transcytosis of various phage families through colonic (T84 and Caco2), lung (A549), and liver (Huh7) epithelial cell lines. While the percentage of transcytosed phages varied between families, transcytosis was preferred in the apical to basal direction in all cases [133]. Microscopy and cellular fractionation revealed that phages were internalized by endocytosis and were trafficked through the Golgi apparatus before being released basally [133]. Inhibitors of endocytosis block the uptake of natural and engineered phages, suggesting that this could be a prominent mode of access to eukaryotic epithelial cells [134–136]. Current estimates suggest that approximately 2 × 10^12^ phages inhabit the human colon [133, 137, 138]. Based on these numbers, Nguyen et al. speculated that over 30 billion daily transcytosis events occur through the epithelium. This nonspecific mode of uptake is likely a powerful mechanism that accounts for the presence of phages systemically in healthy individuals [133]. Another possible mechanism for phages crossing the epithelium barrier includes the Trojan horse theory, whereby a phage-infected bacterium is taken up by an epithelial cell, although there currently is no evidence of this [139, 140].

### 4.2. Immune Recognition and Responses to Phages

After crossing the epithelium, it is hypothesized that phages drain into the lymphatic system where they interact with circulating dendritic cells (DCs) and macrophages to stimulate cytokine production and generate humoral immune responses (Figure 2(b)). The vast genetic diversity of phages in the human gut reflects wide differences in phage morphologies, replication cycles, and structural proteins. Consequently, the direct interactions between phages and the immune system are complex and specific between the phage and the immune cell of interest. Still, most data suggest that phages have either weak proinflammatory or immunomodulatory effects. In a study where 5 × 10^8^ pfu · ml^−1^ T4 phages were individually administered to bone marrow-derived dendritic cells, human plasma, or healthy mice, no increase in cytokine production or production of reactive oxygen species (ROS) was detected [141].

In another study, Miedzybrodzki et al. found that the T4 phage was immunomodulatory by reducing ROS production [142]. Indeed, a preparation of T4 phages inhibited ROS production from peripheral blood polymorphonuclear leukocytes (PMNs) stimulated by LPS or several *E. coli* strains [142]. These findings are all in agreement with the observations that T4 phages reduce immune cell infiltration of an allogeneic skin transplant and reduce T cell proliferation and NF-*κ*B activation in mouse models [143]. Similarly, it has been shown that NF-*κ*B activity can been be modulated by the *Staphylococcus aureus* phage, vB_SauM_JS25. In LPS-stimulated MAC-T bovine mammary epithelial cells, vB_SauM_JS25 inhibited production of several proinflammatory cytokines and inhibited NF-*κ*B signaling [144]. The abilities of T4 and *S. aureus* phages to inhibit the NF-*κ*B pathway could represent a common mechanism for phages to elicit anti-inflammatory responses. The systemic presence of phages in the human body and their anti-inflammatory properties could be important in modulating immune responses and limiting autoimmune or inflammatory disorders [145]. Indeed, when phages infect their bacterial hosts in the bloodstream, dampening the immune response would be important because of the massive release of PAMPs resulting from bacterial lysis.

This perspective on phage-immune system interactions is likely oversimplistic, as there is substantial evidence that certain phages or phage communities can elicit proinflammatory immune responses. For example, *S. aureus* phage A20/R was shown to mediate costimulatory activity in splenocyte proliferation and induce production of the proinflammatory cytokine, IL-6 [146]. There are also examples of phage nucleic acids stimulating antiviral immune responses by activating Toll-like receptors (TLRs) [139]. The archetype filamentous phage M13 was shown to stimulate interferon production and protect mice against tail lesions caused by the vaccinia virus [147]. Eriksson et al. found that the use of tumor-specific phages led to a B16 tumor regression resulting from neutrophil infiltration [148]. Using MyD88-deficient mice, the authors found that this immune activation was dependent on phage induction of TLRs, which causes polarization of tumor-associated macrophages (TAM) to a proinflammatory M1 state [148].

Importantly, there is now increasing evidence that these proinflammatory interactions between immune cells and phages could be relevant in immunological disorders. A recent study showed that a cocktail of 3 *E. coli* phages isolated from IBD patients increased the proportion of CD4+ T cells, CD8+ T cells, and IFN-*γ*-producing T cells in Peyer's patches of germ-free mice [136]. The authors found that this T cell-mediated IFN-*γ* production was dependent on interactions with DCs [136]. Using an *in vitro* approach, they found that these phages were endocytosed by DCs and interacted with TLR9 within endosomes, important sensors implicated in immunity against eukaryotic viruses [136]. The authors then went on to demonstrate that specific pathogen-free mice given this phage cocktail had exacerbation of dextran sodium sulfate- (DSS-) induced colitis and increased levels of TLR9-mediated production of IFN-*γ* [136]. They further assessed that DCs cultured with VLPs isolated from UC patients stimulated higher IFN-*γ* production in comparison to healthy controls *in vitro*, suggesting that certain phage communities might generate more proinflammatory responses [136]. Dysbiosis of phage communities has been correlated with several inflammatory diseases [50–53]. In humans and in a T cell mouse model of colitis, increased abundance of *Caudovirales* has been observed relative to household controls. While it is unclear whether this dysbiosis could drive the development of these disorders, the proinflammatory potential of phage-immune cell interactions should be considered when studying these diseases and developing therapeutics.

Adding to the complexity of the phage-host immune crosstalk, there are several examples of phages which simultaneously elicit pro- and anti-inflammatory responses. Van Belleghem et al. analyzed the expression profiles of 12 immune-related genes in blood monocytes after individual exposure to a *S. aureus* phage and several *Pseudomonas aeruginosa* phages [149]. After exposure to each of these phages, genes involved in both pro- and anti-inflammatory immunological pathways were activated in the peripheral blood monocytes. For instance, the induction of the proinflammatory cytokines IL1*α* and IL1*β* coincided with induction of the IL1 receptor antagonist, which reduces proinflammatory responses [149]. These findings are in agreement with the discovery that filamentous *Pseudomonas* prophages (Pf4) are recognized by TLR3, resulting in transcription of type-1 interferons (IFN), often responsible for clearance of eukaryotic viral infections [150, 151]. This increase in type-1 IFN inhibited TNF, allowing for *P. aeruginosa* to persist and cause infection [150]. In support of their findings, a majority of *P. aeruginosa-*infected wounds contain detectable Pf4 [150].

### 4.3. Antibody Response to Phages

Once across the epithelial layer, neutralizing antibodies could limit further body-wide phage dissemination (Figure 2(b)). Immunization studies have indeed shown that humoral immune responses to phages can be generated. Some early investigations showed that various phages administered to animals or humans can generate specific neutralizing antibody responses [152–154]. It has long been thought that only antibodies that bind to the tail fibre region and inhibit phage-host interactions could abrogate phage infectivity. However, several studies demonstrate that phage capsid proteins, including the T4 highly antigenic outer capsid protein (Hoc), can generate antibody responses [155]. Dąbrowska et al. found that antibodies generated against T4 phages specific to the phage surface proteins, gp23 and Hoc, decreased phage activity [156]. The authors suggested that the antibodies generated against head proteins could prevent phage activity by causing aggregation of phage particles or interaction with the immune complement system to destabilize phage capsids or sterically inhibit phage-bacterial interactions [156].

The production of antiphage antibodies is not exclusive to individuals immunized with phages. The detection of antibodies specific to the T4 phage in the serum of animals with no history of immunization was discovered by Jerne in 1956 [152]. More recently in a group of 50 healthy human volunteers with no prior exposure to phage therapy or immunization, 81% had antibodies in their serum specific to the T4 phage [156]. These data support the idea that natural phage communities could indeed transcytose the epithelium and elicit a humoral immune response.

## 5. Considerations for Phage Therapy

Given the alterations in phage and microbial communities that are observed in a number of inflammatory diseases, there is a potential to use phages to manipulate the microbiota towards a less proinflammatory composition. The long-term stability of phages in the gut and their capacity to alter bacterial hosts offer promise for the design of narrow or whole community phage cocktails that target members of the microbial communities implicated in disease. Before these therapeutic cocktails become a reality, we need to understand phage-host interactions that occur in the context of health and how they differ in inflammation. The contributions of prophage induction to changes in bacterial and phage communities, the host range of phages in the gut, phage-phage interactions, and whether predator-prey dynamics shift during inflammation are questions that still remain unanswered.

Nevertheless, we are beginning to characterize the diversity of phages in the human gut and understand how they might interact in various ways with the immune system. The ability for phages to cross the epithelium barrier and stimulate immune responses has strong implications for the effectiveness of phage therapy. The production of antibodies against phages and their proinflammatory potential raise questions for the efficacy and safety of such approaches. Understanding which phage taxa elicit pro- or anti-inflammatory responses will go a long way in determining which phages might be appropriate for a given condition. Much of the data summarized here on the direct interactions between phages and the immune system focus on a narrow group of phages, often in isolated settings. Elucidating these interactions at a whole community level will help us appreciate the degree to which phages influence immune responses in the human body. Either through their abilities to regulate bacterial populations or through their potential to directly stimulate immune responses, it is clear that phages are active and dynamic players in human health and cannot remain unconsidered in gut microbiome studies.
